# Effect of PKC/NF-*κ*B on the Regulation of P2X_3_ Receptor in Dorsal Root Ganglion in Rats with Sciatic Nerve Injury

**DOI:** 10.1155/2020/7104392

**Published:** 2020-09-17

**Authors:** Xue Li, Jie Yuan, Xuan Yu, Qin Zhang, Bangyong Qin

**Affiliations:** ^1^Department of Pain Medicine, The Affiliated Hospital of Zunyi Medical University, 149 Dalian Road, Huichuan District, Zunyi, Guizhou 563003, China; ^2^Department of Anesthesiology, The Second Affiliated Hospital of Zunyi Medical University, Zunyi 563000, China

## Abstract

**Background:**

Protein kinase C (PKC), nuclear factor-kappa B p65 (NF-*κ*B p65), and P2X_3_ receptor (P2X_3_R) play significant roles in the sensitization and transduction of nociceptive signals, which are considered as potential targets for the treatment of neuropathic pain. However, the mechanisms and relationships among them have not been clearly clarified.

**Methods:**

80 rats were randomized and divided into 10 groups (*n* = 8). Sciatic chronic constriction injury (CCI) rats were intrathecally administered with bisindolylmaleimide I (GF109203X), a PKC-selective antagonist once a day, or pyrrolidine dithiocarbamate (PDTC), an NF-*κ*B inhibitor twice a day. Sham-operated rats were intrathecally administered with saline. Thermal withdrawal latency (TWL) and mechanical withdrawal threshold (MWT) were evaluated in all the groups before CCI operation (baseline) and on the 1st, 3rd, 7th, 10th, and 14th day after CCI operation. Protein levels of p-PKC*α*, p-NF-*κ*B p65, and P2X_3_R were analyzed in the CCI ipsilateral L_4–6_ dorsal root ganglions (DRGs).

**Results:**

Intrathecal injection of GF109203X or PDTC alleviated the TWL and MWT in the following 2 weeks after CCI surgery. The protein levels of p-PKC*α*, p-NF-*κ*B p65, and P2X_3_R in the ipsilateral DRGs significantly increased after CCI operation, which could be partly reversed by intrathecal administration of GF109203X or PDTC.

**Conclusion:**

The upregulation of p-PKC*α*, p-NF-*κ*B p65, and P2X_3_R expression in the DRGs of CCI rats was involved in the occurrence and development of neuropathic pain. Phosphorylated PKC*α* and phosphorylated NF-*κ*B p65 regulated with each other. Phosphorylated NF-*κ*B p65 and PKC*α* have a mutual regulation relationship with P2X_3_R, respectively, while the specific regulatory mechanism needs further research.

## 1. Introduction

Neuropathic pain refers to chronic pain caused by disease or damage to the central or peripheral nervous system. About 6.9% to 10% of the world's undefined population suffers from neuropathic pain [1]. There are four characteristic symptoms: spontaneous pain, hyperalgesia, abnormal pain, and sensory disturbance. Because of its complex pathogenesis, the exact diagnosis is difficult and the treatment effect is unsatisfied. Neuropathic pain has a great influence on the patient's undefined physical and mental health and quality of life.

Dorsal root ganglions (DRGs) contain numerous primary sensory neurons [2]. DRGs are located in the hole near the pedicle of the vertebral arch [3]. They are vital components of integrating and transmitting nociceptive signals from peripheral nerves to the central nervous system [4]. Sommer [5, 6] showed that DRGs played a key role in the transmission and regulation of chronic pain. DRG neurons contain multiple ion channels and receptors, and the changes of ion channels and receptors cause neuropathic pain when noxious stimuli occur [7]. The excitatory increase of DRG neurons is closely related to the occurrence and maintenance of neuropathic pain [2].

Protein kinase C (PKC) is a multifunctional serine and threonine kinase family composed of a single peptide chain. It is widely distributed in the peripheral and central nervous systems. As one of the second messengers in the cell, PKC regulates a variety of receptors, ion channels, and cell functions, such as proliferation, differentiation, and apoptosis. Previous studies have shown that PKC plays an important role in the conduction of nociceptive stimulation and is closely related to pain [8]. Wu et al. suggested that PKC may be a major regulator of peripheral and central sensitization [9]. PKC participates in the regulation of pain by transmitting noxious stimulus signals generated after peripheral nerve or tissue damage to the dorsal horn of the spinal cord in primary afferent neurons [10]. Activation of PKC can increase the excitability of sensory neurons, which promotes the occurrence and development of pain, and inhibiting the activation of PKC can reduce hyperalgesia [11].

Nuclear factor-kappa (NF-*κ*B) is a key transcription factor in the regulation of various gene expressions and is involved in the regulation of the expression of many pain gene media. NF-*κ*B is involved in the pathophysiological processes of inflammation, immunity, stress, and the like and is closely related to neuropathic pain [12]. The NF-*κ*B family includes five members (p50, p52, p65, c-Rel, and Rel-B) [13]. Within the nervous system, NF-*κ*B generally consists of a p50/p65 heterodimer [14]. Under normal conditions, the NF-*κ*B dimers form complexes with members of a family of inhibitors (inhibitor of *κ*B and I*κ*B), which mask the nuclear localization signal of NF-*κ*B and retain it in an inactive state within the cytoplasm. The phosphorylation of I*κ*B by the I*κ*B kinase triggers inhibitor degradation, releasing NF-*κ*B and promoting its nuclear translocation and the modulation of gene expression [13]. Souza et al. have shown that PKC activates NF-*κ*B in DRGs to maintain persistent inflammatory pain in rats [15]. After sciatic chronic constriction injury (CCI) operation, levels of NF-*κ*B p65 in DRGs increased, leading to the decrease of hot pain, cold pain, and mechanical pain threshold, which could be partly reversed by intrathecal injection of pyrrolidine dithiocarbamate (PDTC) [16]. Therefore, NF-*κ*B participates in the occurrence and development of neuropathic pain.

ATP has been reported to be an important mediator of nociception and acts through P2X and P2Y, two different receptor families [17]. P2X receptors are important nociceptive candidates [18]. So far, seven P2X subunits, namely, P2X1–7, have been identified [17]. Previous studies have demonstrated that the P2X receptors, especially the P2X_3_ receptor (P2X_3_R), were closely related to the generation and transmission of pain signals [19]. P2X_3_R was reported to be localized predominantly on small-to-medium diameter C-fiber nociceptive sensory neurons within the DRGs [20, 21]. The high expression of P2X_3_R in DRGs plays a crucial role in the development and occurrence of pain [22]. In the diabetic neuropathic pain model, intrathecal injection of 5-[[[(3-Phenoxyphenyl)methyl][(1S)-1,2,3,4-tetrahydro-1-naphthalenyl]amino]carbonyl] 1,2,4-benzenetricarboxylic acid sodium salt hydrate (A-317491), a specific antagonist of P2X_3_R, can reduce hyperalgesia [23]. Therefore, P2X_3_R is likely to participate in the generation and transmission of neuropathic pain.

PKC could sensitize P2X_3_R [24]. Phosphorylation of PKC could promote the activation of P2X_3_R and cause neuropathic pain. Inhibition of the expression of phosphorylation PKC could inhibit the expression of P2X_3_R, thus alleviating the pain [25]. The inhibitor of PKC could inhibit P2X_3_ R-mediated currents, while the activator of PKC could activate P2X_3_ R-mediated currents [26]. The activation of P2X_3_R could lead to calcium influx, which causes the activation of PKC [27]. NF-*κ*B p65 and P2X_3_R were coexpressed in DRG neurons. NF-*κ*B p65 regulated the expression of P2X_3_R at a transcriptional level which could adjust the function of P2X_3_R by regulating the expression of P2X_3_R, which led to diabetic pain hypersensitivity. Intrathecal injection of NF-*κ*B p65 specific inhibitor or specific siRNA vector (LV-p65) targeting the NF-*κ*B p65 gene could reduce pain behavior by inhibiting the activity and expression of P2X_3_R [23]. Besides, activated PKC causes the phosphorylation and degradation of I*κ*B, which is an inhibitory protein of NF-*κ*B, leading to the activation of NF-*κ*B [14, 28]. Therefore, we infer that PKC/NF-*κ*B may be involved in the regulation of P2X_3_R on neuropathic pain in rats with sciatic nerve injury.

## 2. Methods

### 2.1. Ethical Approval and Animal Preparation

Animal protocols were approved by the Experimental Animal Care and Use Committee of Zunyi Medical University (approval number ZMC2013-0009). Eighty male 7-week-old Sprague Dawley rats (180–200 g) were purchased from the Changsha Tianqin Biotechnology (Hunan, China) and housed under a standard 12-hour light/dark cycle with free access to food and water. All animals were housed at least 1 week before our experiments. All the procedures complied with the standard protocols or Guide for the Care and Use of Laboratory Animals (8th edition, 2011).

### 2.2. Placement of Intrathecal Catheter and Drug Administration

Rats were anesthetized intraperitoneally with 1% Nembutal (40 mg/kg). Procedures of the intrathecal catheter were conducted as reported previously [29]. A 2 cm skin incision was made, and tissues were bluntly dissected to expose the spinal dura mater. Then, the spinal dura mater was pierced with a syringe needle, and a prepared polyethylene catheter was carefully implanted into the lumbar enlargement and fixed with sutures. After 24 hours, neurological functions were evaluated, and the rats with neurological deficiency were excluded. If the catheter was placed in the subarachnoid space successfully, an injection of 10 *μ*L 2% lidocaine through the intrathecal catheter could induce paralysis of the hind limb within 30 s. Administration of 20 wu·units/d antibiotics was used to prevent animals from infection after the operation for 3 days. PKC inhibitor bisindolylmaleimide I (GF109203X) and NF-*κ*B inhibitor pyrrolidine dithiocarbamate (PDTC) were purchased from Sigma-Aldrich (Shanghai, China). In the following two weeks after CCI surgery, GF109203X was given intrathecally once a day in the GF group, and PDTC was given intrathecally twice a day in the PDTC group. In the Sham group and CCI group, 20 *μ*L saline was applied intrathecally. In the DMSO group, 20 *μ*L DMSO was applied intrathecally; 10 *μ*L of 0.36 *μ*g [30] GF109203X (studies showed that this concentration has a good analgesic effect [30]) + 10 *μ*L DMSO or 10 *μ*L of 20 *μ*g [31] PDTC (previous studies have shown that this concentration had a good analgesic effect [16]) + 10 *μ*L saline were applied intrathecally in the GF group or PTDC group, respectively.

### 2.3. CCI Model Establishment

CCI surgery was performed 1 day after the successful placement of the intrathecal catheter. All surgical procedures were conducted as reported by Bennett and Xie [32]. Rats were intraperitoneally anesthetized with 1% Nembutal (40 mg/kg), and the right sciatic nerve behind the femur was bluntly dissected and fully exposed. Three to four ligatures were made with 4–0 silk sutures with an appropriate degree of constriction which induced slight tremor of the calf muscle but did not arrest the blood flow. For the rats in the Sham group, the sciatic nerve was just bluntly dissected through the biceps femoris, without the management of ligatures. Rats in different groups were raised separately.

### 2.4. Pain Threshold Evaluation

In all groups, the pain threshold was evaluated before CCI surgery (baseline) and 1st, 3rd, 7th, 10th, and 14th days after CCI surgery, respectively. Thermal withdrawal latency (TWL) was measured using a plantar thermal stimulator (IITC Life Science, USA) with a 20% energy value. Evaluation of TWL was performed when rats adapted to the testing environment and kept still. The time of duration from the beginning of the beam to the appearance of the first withdrawal movement on the right hind paw was recorded. The maximum time of the beam was 30 s so as to prevent tissue injury. Five measurement indicators were obtained from each rat with 5 min intervals, and the mean of these indicators was defined as TWL.

Mechanical withdrawal threshold (MWT) was evaluated by using an electronic von Frey plantar aesthesiometer (IITC Life Science, USA). Continuous graded mechanical stimulation was applied vertically on the right hind paw, and the test was completed until the rats lifted their hind paws. Five measurements were taken for each rat with 5 min intervals, and the mean of these indicators was defined as MWT.

### 2.5. Western Blotting

After behavioral tests, rats in all groups were intraperitoneally anesthetized with 1% Nembutal (40 mg/kg) and sacrificed on the 7th or 14th day after CCI surgery. Attal et al. showed that neuropathic pain symptoms begin to appear 2–6 days after CCI surgery, and symptoms peak at 2 weeks [33]. The ipsilateral L_4–6_ DRG tissue was collected. About 30 mg of tissue was homogenized in RIPA lysate with PMSF (Shanghai, China). The homogenate was centrifuged at 12,000 g/min at 4°C. The supernatant was collected, and the protein concentrations were determined using the BCA assay (Shanghai, China). Protein samples were successively loaded into each well of a 10% SDS/PAGE gel and followed by 2 hours of electrophoresis. Then, proteins were electrotransferred to a polyvinylidene difluoride membrane with a 60-minute transfer time. The film was fully immersed in 5% BSA-TBST, and it was shaken lightly at room temperature for 60 min. The following primary antibodies were added: rabbit anti-PRKCA polyclonal antibody (1 : 4000, D151256, Shanghai, China), rabbit polyclonal antibody against NF-*κ*B p65 (1 : 2000, D220135, Shanghai, China), and rabbit anti-P2RX3 polyclonal antibody (1 : 2000, 17843-1-AP, Wuhan, China). It was shaken at 4°C for the whole night. Then, the membranes were incubated with HRP-conjugated secondary antibodies at room temperature for 1 hour. The immunoreactive protein bands were detected by enhanced chemiluminescence with an ECL kit (Shanghai, China). Rabbit anti-beta-actin polyclonal antibody (1 : 3000, D110001, Shanghai, China) was used as an internal reference antibody. The relative optical density of the protein bands was measured, and protein levels were normalized to *β*-actin. Data are expressed as mean ± SD of the target/*β*-actin.

### 2.6. Statistical Analysis

Statistics were performed using GraphPad Prism (version 7.0, GraphPad Software, USA). All data were expressed as mean ± SD. Statistical analysis was performed using the independent sample *T*-test. *P* < 0.05 was considered statistically significant.

## 3. Results

### 3.1. Inhibition of PKC or NF-*κ*B Increased Thermal and Mechanical Pain Thresholds in CCI Rats

In the CCI group and DMSO group, neuropathic pain behaviors such as back flexion, licking, and toe closing were observed on the first day after CCI surgery, while motor function and autonomy were intact. For the rats in the GF109203X group and PDTC group, neuropathic pain behaviors described above were also observed on the first day after CCI surgery, which were significantly lighter than those in CCI and DMSO groups. For rats in the Sham group, neuropathic pain behaviors did not occur after intrathecal injection of normal saline.

**Figure 1:**
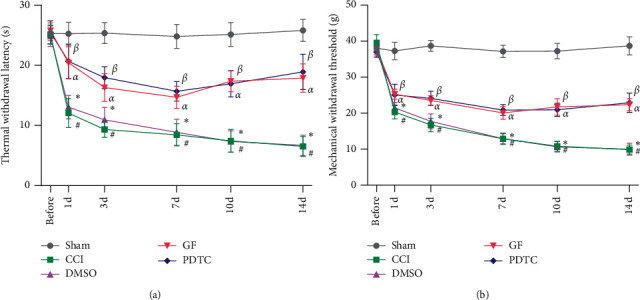
The thermal withdrawal latency and mechanical paw withdrawal threshold in chronic constriction injury (CCI) rats significantly decreased in the CCI group and DMSO group compared with the Sham group after CCI surgery. Inhibiting PKC and NF-*κ*B increased the thermal withdrawal latency and mechanical paw withdrawal threshold in CCI rats. Data are expressed as mean ± SD (*n* = 16 for each group). Statistical analysis was conducted by the one-way ANOVA tests and the S-N-Q test. ^#^*P* < 0.05, ^*∗*^*P* < 0.05 vs. Sham group, ^*α*^*P* < 0.05, ^*β*^*P* < 0.05 vs. CCI group. ^*α*^*P* < 0.05 vs. CCI + GF109203X group.

Compared with the Sham group, the thresholds of thermal withdrawal latency and mechanical paw withdrawal decreased significantly in the CCI and DMSO groups since the first day after CCI surgery. Compared with the CCI group, inhibiting PKC with GF109303X or NF-*κ*B with PDTC significantly increased thermal withdrawal latency and mechanical paw withdrawal threshold after CCI surgery. Compared with the DMSO group, inhibiting PKC with GF109303X contributed to an obvious increase of both thermal withdrawal latency and mechanical paw withdrawal threshold after CCI surgery (Figures 1(a) and 1(b)).

### 3.2. PKC Participated in the Neuropathic Pain via Regulating NF-*κ*B or P2X_3_R

In order to explore whether PKC participated in the neuropathic pain regulation through NF-*κ*B and P2X_3_R, we detected p-PKC*α*, p-NF-*κ*B p65, and P2X_3_R protein levels and retested the pain thresholds after inhibiting PKC.

**Figure 2:**
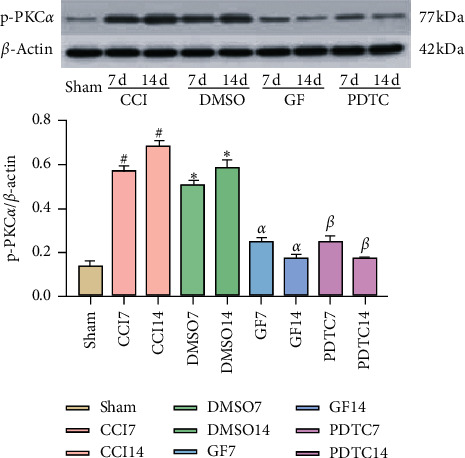
Inhibiting PKC or NF-*κ*B reduced CCI-induced p-PKC*α* overexpression in DRGs. Data are expressed as mean ± SD and analyzed with one-way ANOVA tests and the S-N-Q test. ^#^*P* < 0.05, ^*∗*^*P* < 0.05 vs. Sham group, ^*α*^*P* < 0.05, ^*β*^*P* < 0.05 vs. CCI group, ^*α*^*P* < 0.05 vs. DMSO group.

**Figure 3:**
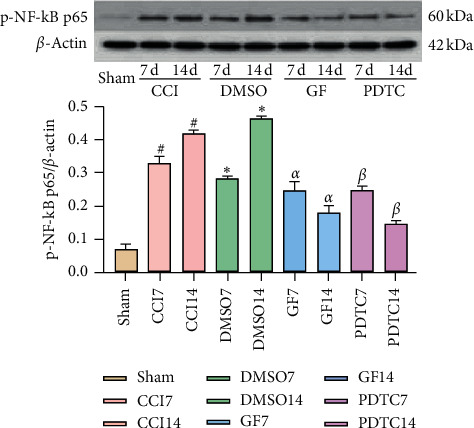
Inhibiting PKC and NF-*κ*B reduced CCI-induced p-NF-*κ*B p65 overexpression in DRGs. Data are expressed as mean ± SD and analyzed with one-way ANOVA tests and the S-N-Q test. ^#^*P* < 0.05, ^*∗*^*P* < 0.05 vs. Sham group, ^*α*^*P* < 0.05, ^*β*^*P* < 0.05 vs. CCI group, ^*α*^*P* < 0.05 vs. DMSO group.

**Figure 4:**
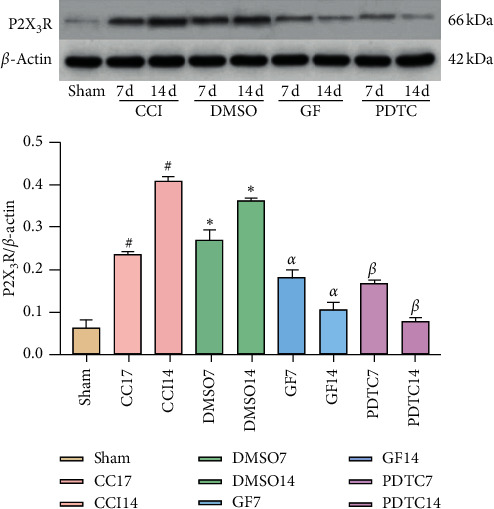
Inhibiting PKC and NF-*κ*B reduced CCI-induced P2X_3_R overexpression in DRGs. Data are expressed as mean ± SD and analyzed with one-way ANOVA tests and the S-N-Q test. ^#^*P* < 0.05, ^*∗*^*P* < 0.05 vs. Sham group, ^*α*^*P* < 0.05, ^*β*^*P* < 0.05 vs. CCI group, ^*α*^*P* < 0.05 vs. DMSO group.

Compared with the Sham group, levels of p-PKC*α*, p-NF-*κ*B p65, and P2X_3_R significantly increased on the 7th and 14th day after CCI surgery in the CCI and DMSO groups while levels of the three proteins in the GF109203X group were significantly lower than those in CCI and DMSO groups on the 7th and 14th day after CCI surgery (Figures 2–4).

### 3.3. NF-*κ*B Participated in the Neuropathic Pain via Regulating P2X_3_R

In order to prove whether NF-*κ*B participated in the neuropathic pain regulation through P2X_3_R, we detected NF-*κ*B p65 and P2X_3_R expression and retested the pain thresholds after inhibiting NF-*κ*B.

Compared with the Sham group, levels of NF-*κ*B p65 and P2X_3_R significantly increased on the 7th and 14th day after CCI surgery in the CCI group, while levels of the two proteins in the PDTC group were significantly lower than those in the CCI group on the 7th and 14th day after CCI surgery (Figures 2–4).

## 4. Discussion

The effective treatment of neuropathic pain remains challenging. Neuropathic pain, a type of chronic pain as a result of direct central or peripheral nerve damage, is associated with significant quality of life and functional impairment. Its underlying mechanisms remain unclear. Because of the complicated mechanism of neuropathic pain, most clinical treatment methods are nonspecific, and the therapeutic effect is unsatisfactory.

There are 10 subtypes of PKC, which are *α, β*I, *β*II, *γ*, *δ*, *ε*, *θ*, *η*, *ζ*, and *λ* [34]. As the second messenger in the cell, PKC is closely related to inflammatory pain and neuropathic pain. Velazquez et al. [35] reported that PKC*α* and *ε* were mainly involved in peripheral nociception, which could transmit nociceptive signals from the injured surrounding area to the spinal dorsal horn, while PKC*γ* plays an important role in central nociception. In the CFA-induced inflammatory pain model, the expression of the p-PKC protein in the rat contact nucleus (CSF-CN) was higher than that in the blank group. Lateral ventricle injection GF109203X reduced the level of p-PKC protein expression in CSF-CN and relieved the hyperalgesia [36]. Some studies have shown that PKC can regulate the excitability of DRG neurons by regulating the Nav1.3 channel in the spinal nerve ligation (SNL) model of neuropathic pain [37]. The results of Zhou et al. showed that the expression of p-PKC in L_4–6_ DRGs was significantly increased in painful diabetic neuropathy (PDN). Intra-abdominal injection of phorbol-12-myristate-13-acetate (PMA), an activating agent of PKC, could block the analgesic effect of electroacupuncture [25]. Inhibition of PKC expression in the DRGs of diabetic rats can reduce hyperalgesia in rats [38]. In the results of this experiment, the expression of p-PKC*α* in the DRGs of CCI and DMSO groups increased, and the pain thresholds of rats decreased. The expression of p-PKC*α* in the DRGs decreased, and the pain thresholds of rats increased after intrathecal injection of the GF109203X, a selective inhibitor of PKC. It is suggested that PKC*α* may cause and maintain neuropathic pain.

NF-*κ*B is a key transcription factor, which can promote the expression of inflammatory factors, including tumor necrosis factor-*α* (TNF-*α*), interleukin-1*β* (IL-1*β*), and IL-6, etc., which is involved in neuropathic pain or inflammatory pain [39]. Inhibition of NF-*κ*B activation inhibits the expression of inflammatory substances [40]. The effects of mechanical and thermal hyperalgesia and proinflammatory cytokines could be significantly inhibited after intrathecal injection of NF-*κ*B specific inhibitor PDTC [41]. In the model of neuropathic pain induced by CCI, the expression of NF-*κ*B in the L_4–6_ segment of the spinal cord could be detected, and the inhibition of the NF-*κ*B pathway could alleviate the allergic reaction of neuropathic pain [42]. The results of previous studies showed that the expression of NF-*κ*B p65 in DRGs of CCI rats increased, and the expression of NF-*κ*B p65 in DRGs downregulated after intrathecal injection PDTC, a selective inhibitor of NF-*κ*B, and the hyperalgesia symptoms of CCI rats significantly reduced [16]. In the results of this experiment, the expression of p-NF-*κ*B p65 in the DRGs of CCI and DMSO groups increased, and the pain thresholds of rats decreased. The expression of p-NF-*κ*B p65 in the DRGs decreased, and the pain thresholds of rats increased after intrathecal injection the PDTC, a selective inhibitor of NF-*κ*B, which indicated that NF-*κ*B p65 was involved in the production and maintenance of neuropathic pain. Besides, activated PKC causes the phosphorylation and degradation of I*κ*B, which is an inhibitory protein of NF-*κ*B, leading to the activation of NF-*κ*B [14, 28]. The research of Zhao et al. showed that PKC-NF-*κ*B participated in the increase of Nav1.8 current density induced by chemokine ligand 2 (CCL2), which caused inflammatory hyperalgesia by promoting the phosphorylation of Nav1.8 in DRGs and its expression [43]. Inhibition of the activation of PKC in DRGs could inhibit NF-*κ*B p65 subunit translocation, thereby reducing inflammatory hyperalgesia in rats [15]. In the results of this experiment, the expression of p-PKC*α* and p-NF-*κ*B p65 in the DRGs of rats decreased after intrathecal injection GF109203X. Therefore, we speculate that p-PKC*α* may be involved in neuropathic pain by regulating the activation of NF-*κ*B.

P2X_3_R is an important nociceptive receptor. Accumulated evidence suggests that P2X_3_R in DRG neurons plays a major role in mediating chronic pain associated with nerve injury. It plays an important role in inflammatory pain and neuropathic pain [44, 45]. After the injection of CFA into the right posterior claw of rats, the expression of P2X_3_R in DRGs was upregulated, and the thresholds of heat pain were decreased. The expression of P2X_3_R in DRGs was downregulated, and the thresholds of heat pain were increased after electroacupuncture (EA) treatment. Also, agonist P2X_3_R could decrease the analgesic level of EA [46]. After CCI surgery, the expression of P2X_3_R protein and mRNA on DRGs increased, while the thermal pain thresholds and mechanical pain thresholds decreased in rats. The expression of P2X_3_R protein and mRNA was downregulated after intrathecal injection of P2X_3_R specific antagonist A-317491, and the thermal pain thresholds and mechanical pain thresholds were increased [47, 48]. PKC could sensitize P2X_3_R [24]. Phosphorylation of PKC could promote the activation of P2X_3_R and cause neuropathic pain. Inhibition of the expression of phosphorylation PKC could inhibit the expression of P2X_3_R, thus alleviating the pain [25]. It is reported that PKC*α* is abundantly expressed in DRGs, and p-PKC*α* was involved in pain. The upregulation of p-PKC*α* expression led to the sensitization of P2X_3_R, causing the occurrence and development of pain [49, 50]. Besides, NF-*κ*B p65 and P2X_3_R were coexpressed in DRG neurons. NF-*κ*B p65 regulated the expression of P2X_3_R at a transcriptional level which could adjust the function of P2X_3_R by regulating the expression of P2X_3_R, which led to diabetic pain hypersensitivity. Intrathecal injection of NF-*κ*B p65 specific inhibitor or specific siRNA vector (LV-p65) targeting the NF-*κ*B p65 gene could reduce pain behavior by inhibiting the activity and expression of P2X_3_R [23]. The research of Brown et al. showed that calcium ion can promote the activation of NF-*κ*B, thereby promoting the release of inflammatory factors [51]. Therefore, we speculate that P2X_3_R may regulate NF-*κ*B p65 through calcium ion. In the results of this experiment, the expressions of p-PKC*α*, p-NF-*κ*B p65, and P2X_3_R were downregulated after intrathecal injection of GF109203X. We speculate that PKC may regulate the expression of NF-*κ*B through P2X_3_R, or PKC may regulate the expression of P2X_3_R through NF-*κ*B. However, after intrathecal injection of PDTC, the expressions of p-NF-*κ*B p65, P2X_3_R, and p-PKC*α* were also downregulated, indicating that NF-*κ*B could regulate the expression of P2X_3_R, and then P2X_3_R promoted the activation of PKC. The possible mechanism is that the activation of P2X3R promotes calcium influx to further activate PKC, thereby participating in neuropathic pain.

## 5. Conclusion

The upregulation of p-PKC*α*, p-NF-*κ*B p65, and P2X_3_R expression in the dorsal root ganglion of CCI rats was involved in the occurrence and development of neuropathic pain. Phosphorylated PKC*α* and phosphorylated NF-*κ*B p65 regulated with each other. Phosphorylated NF-*κ*B p65 and PKC*α* have a mutual regulation relationship with P2X_3_R, respectively, while the specific regulatory mechanism needs further research.

## Data Availability

The experimental data used to support the findings of this study are included within the article.
